# Analysis of Rural Disparities in Ultrasound Access

**DOI:** 10.7759/cureus.25425

**Published:** 2022-05-28

**Authors:** Nicholas J Peterman, Eunhae Yeo, Brad Kaptur, Emily J Smith, Anton Christensen, Edward Huang, Mehmoodur Rasheed

**Affiliations:** 1 Medicine, Carle Illinois College of Medicine, Champaign, USA; 2 Biomedical Engineering, University of Illinois Urbana-Champaign, Champaign, USA; 3 Rheumatology, Carle Foundation Hospital, Champaign, USA

**Keywords:** geospatial analysis, rural, disparities, access, ultrasound

## Abstract

Purpose

This work aims to conduct a geospatial analysis of recent ultrasound access and usage within the United States, with a particular focus on disparities between rural and urban areas.

Methods/Materials

Multiple public datasets were merged on a county level, including US Department of Agriculture economic metrics and Centers for Medicare Services data using the most recent years available (2015-2019). From these databases, 39 total variables encompassing the socioeconomic, health, and ultrasound characteristics of each county were obtained. Current Procedural Terminology (CPT) codes incorporated included ultrasound-guided procedures and diagnostic exams. Three thousand eleven counties were included. The combined dataset was then exported to GeoDa for network-based analysis and to produce map visualizations. To identify statistically significant (p < 0.05) hotspots and coldspots in point-of-care ultrasound (POCUS) prevalence, Moran’s I was used. Choropleth maps were created for visualization. ANOVA was run across the four Moran’s I groups for each of 39 variables of interest.

Results

A total of 30,135,085 ultrasound-related CPT codes were billed to Medicare over 2015-2019, with 26.55% of codes being ultrasound-guided procedures and 73.45% being diagnostic exams. 38.84% of rural counties had access to POC ultrasound compared to 88.56% of metropolitan counties and 74.19% of counties overall. Hotspots of POCUS were in Southern California and the Eastern US (average of 1,441 per 10,000 Medicare members per year). Coldspot areas were seen in the Great Plains and Midwest (average of 7.43 per 10k Medicare members per year). Hotspot clusters, when compared to coldspot clusters, were significantly (p < 0.001) more dense (703.6 to 14.9 people per square mile), more urbanized (3.5 to 7.1 Rural-Urban Continuum (RUC)), more college-educated (25.1% to 20.0%), more likely to have an Emergency Department (ED) visit (725.8 to 616.9 visits per 1,000 Medicare members), more likely to be obese (19.0% to 12.9%), less likely to be uninsured (10.1% to 13.0%), had more Black representation (8.5% to 3.4%), and less Hispanic representation (2.6% to 5.5%).

Conclusions

Ultrasound access and usage demonstrate significant geospatial trends across the United States. Hotspot and coldspot counties differ on several key sociodemographic and economic variables.

## Introduction

Ultrasound is an inexpensive, noninvasive, and rapid imaging diagnostic tool whose utility is well-established in healthcare. It is even considered by some to have become the “visual stethoscope of the 21st century” [1]. Since its first use as a medical diagnostic tool in the 1950s, ultrasound has developed to become compact, instantaneous, and radiation-free [2]. Ultrasound was used initially by emergency medicine physicians at bedside to quickly assess trauma patients; since then its role has expanded into intra-abdominal pathology diagnosis, pregnancy and fetus evaluations, and guidance for techniques requiring needle insertion [1]. Specialized features such as tissue characterization and image segmentation have propelled ultrasound performance in diagnostic investigations [3]. As point-of-care ultrasound (POCUS) allows for rapid evaluation at bedside, numerous protocols have been developed using POCUS to provide swift and structured response guidelines to clinical scenarios such as respiratory failure and cardiogenic shock [4]. POCUS-based diagnostic exam protocols have also been shown to have increased accuracy compared to standard clinical or X-ray-based approaches in respiratory failure assessment [5,6]. The use of intravascular ultrasound to image intermediate coronary stenoses has been shown to improve outcomes in patients receiving percutaneous coronary intervention [7]. Ultrasound’s usage in other clinical practices and healthcare settings continues to grow rapidly.

As with many other facets of healthcare, there is unfortunately a disparity between access to ultrasound between rural and urbanized areas in the United States [8-10]. Historically, rural communities have experienced poorer health outcomes compared to urbanized communities [11,12]. Most recent census data shows that about 20% of the US population lives in rural areas, but only 9% of doctors in America are practicing in rural communities [13]. Between 2005 and 2009, the life expectancy for one living in a rural area was 76.7 years versus 79.1 in urbanized areas. This current difference in life expectancy between rural and urbanized areas has only grown over time: The difference was a mere 0.4 years when measured in 1971 [14]. Residents of rural and nonmetropolitan areas also report a considerably higher prevalence of self-assessed fair/poor health, psychological distress, disability, functional limitation, injuries, and hypertension than their metro counterparts [14]. A previously conducted survey of persons living in rural areas demonstrated that the number one healthcare priority of people living in rural areas is access to affordable care, with 76.3% listing it as a priority [13].

Despite the prevalence of ultrasound use, its overwhelming utility, and its inexpensive nature, there exists a disparity between rural and urban ultrasound use. This study aims to compare ultrasound use in rural and urban areas and to evaluate the ease of access to ultrasound technology within rural populations of the United States.

## Materials and methods

This work solely utilized publicly available datasets void of identifying patient information and no attempt was made to merge data specifically in a manner that enhanced the identification of individuals. As such, informed consent and IRB review were waived per federal guideline 45 CFR 46 and institutional policy at the authors’ institutions. Multiple public datasets were merged using Python on a county level for this study, including US Department of Agriculture (USDA) economic metrics and data obtained from the Center for Medicare Services (CMS), which included all Current Procedural Terminology (CPT) codes billed to Medicare organized by provider and geolocation, Medicare demographics, and Medicare chronic disease data [15-18]. All datasets were year matched and averaged across the most recent 5 years of available data (2015-2019). The only exception was the USDA Rural-Urban Continuum (RUC) data, which was from 2013. This dataset ranks the urbanization level of each county on a scale from 1 to 9 with rankings 1-3, 4-7, and 8-9 historically representing metropolitan (metro), urban (but not metro), and rural, respectively [19]. From these databases, 39 total variables encompassing the socioeconomic, health, and ultrasound prevalence of each county in the USA were obtained. Of the 3,109 total counties in the contiguous US, 3,011 were included in the final analysis. In total, 98 counties were excluded due to missing data in any one of the 39 chosen variables.

Medicare billing conventions and individual CPT codes subdivide POCUS as either ultrasound-guided procedures (CPT 76937, 76942, and 76930) or as diagnostic exams (76512, 76604, 76705, 76775, 76830, 76857, 76882, 93308, and 93971). In this analysis, both groups were counted individually and in total, with the total value being used for the majority of subsequent analyses. The total number of services was extracted, scaled to the Medicare population of each county, averaged for all 5 years, and logarithmically scaled. The logarithmic scaling was needed to reduce the skew of the dataset from highly varied POCUS prevalence. A binary variable, access to POCUS, was then created and defined as a county having at least one POCUS CPT code billed over the time period.

The database was then exported to GeoDa, a geospatial analysis program, in order to conduct network-based analysis and produce map visualizations. To identify statistically significant (p <0.05) hotspots and coldspots in POCUS prevalence, the Moran’s I statistic was used. The Moran’s I statistic examines both a county and the average of its neighbors across a variable of interest (in this case log POCUS per 10k Medicare members) and determines if both are statistically significantly higher or lower than the national average, thus disproving the null hypothesis of spatial randomness. If both a county and the average of its neighbors are statistically significantly higher than average, it is considered a spatial cluster and a High-High county. If both a county and the average of its neighbors are statistically significantly lower than average, it is considered a spatial cluster and a Low-Low county. If both a county and the average of its neighbors are statistically significantly higher or lower than average but in opposite directions, then it is a spatial outlier and either Low-High or High-Low. Spatial outliers can be considered transition counties and often demarcate areas where there is a change from rural to urban or urban to rural. Spatial clusters are often colloquially referred to as either hotspots or coldspots. If either the county or the average of its neighbors was not significantly greater or less than the national average, then the county does not reject the null hypothesis and is thus considered not statistically significant.

## Results

Four choropleth maps, or geospatial maps where the color shading is proportional to a variable of interest, were created for visualization of the RUC-derived county classifications, access to POCUS, log POCUS per 10k Medicare members, and the Moran’s I groupings. The dataset was then exported back to Python where an ANOVA was run across the four Moran’s I groups of log POCUS per 10k Medicare members for each of the 39 variables of interest with p < 0.05 for significance.

**Figure 1:**
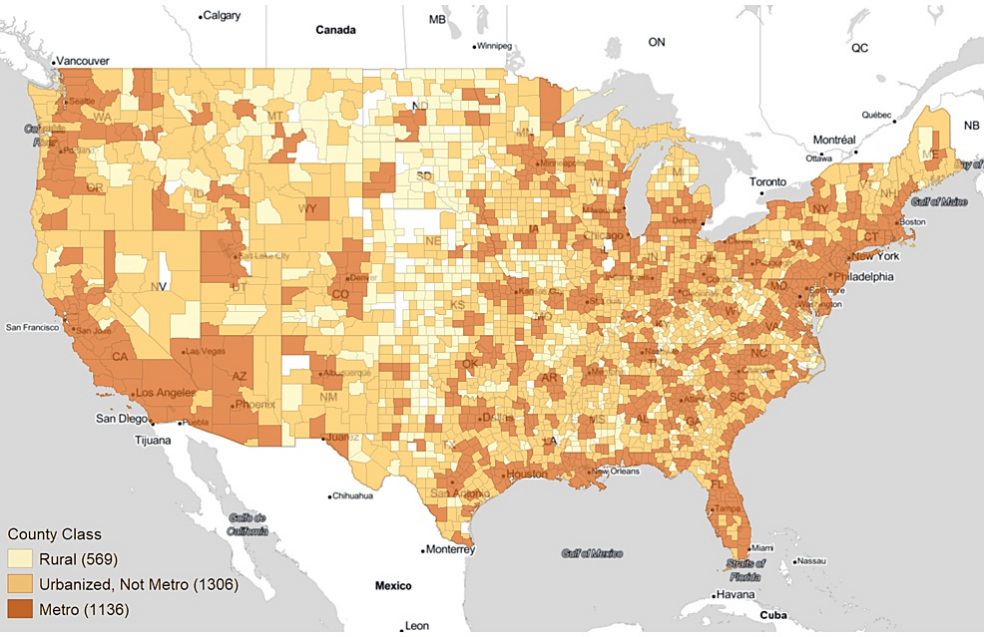
Categorical choropleth map of rural, urbanized, and metro counties Categories were derived from rural-urban continuum classifications of counties: metro = 1-3 RUC, urbanized, not metro = 4-7 RUC, and rural = 8-9 RUC. Counties excluded from analysis are in white. RUC: Rural-Urban Continuum

A simplified RUC score for the urbanization level for each county was first created. The new categories of the metro, urbanized-but-not-metro, and rural are defined as RUC scores of 1-3, 4-7, and 8-9 respectively (Figure 1). RUC was grouped in this manner for improved visualization of the metric and to explicitly define the rural/urban divide used in this analysis. Rural areas are predominantly located in the Great Plains and interspersed among the adjoining Midwest and the Rocky Mountains regions. Urbanized counties tend to surround metro areas throughout the rest of the country.

**Figure 2:**
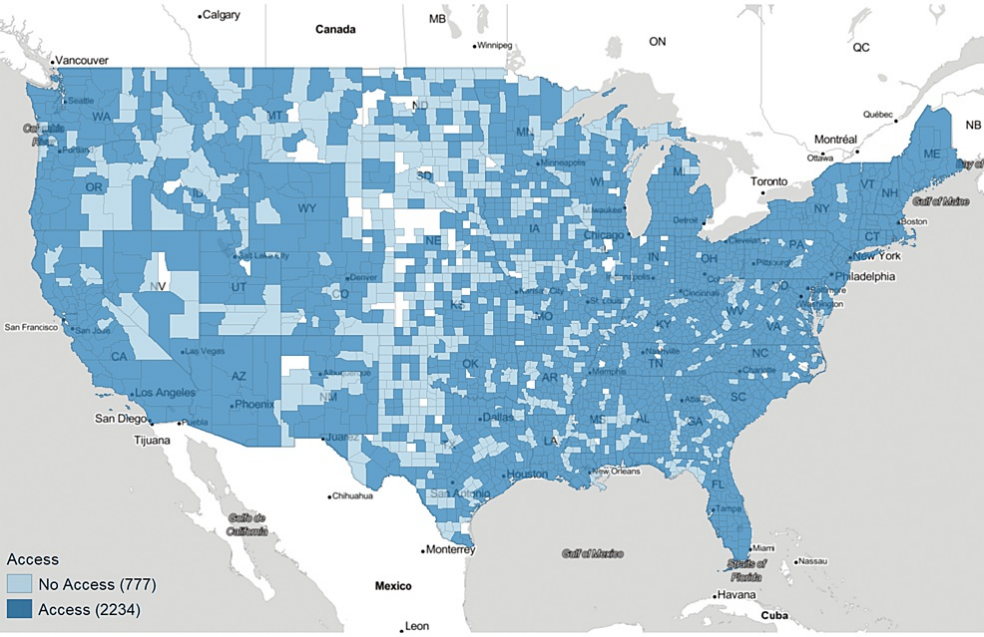
Binary choropleth map of ultrasound access Access is defined per county as having at least one ultrasound procedure billed to Medicare in 2015-2019. Counties excluded from analysis are in white.

A total of 30,135,085 ultrasound-related CPT codes were billed to Medicare over 2015-2019 with 26.55% of codes being ultrasound-guided procedures and 73.45% being diagnostic exams. In our analysis, we defined POCUS access within a county as having at least one POCUS CPT code billed over the period evaluated, which will further be referred to as absolute POCUS or ultrasound access (Figure 2). 38.84% of rural counties had access to POCUS compared to 77.11% of urbanized-but-not-metro counties, 88.56% of metro counties, and 74.19% of counties overall. In secondary analysis, after removing all counties without access, subdivision of the remaining counties into the rural, urbanized-but-not-metro, and metro categories shows that rural POCUS per 10,000 Medicare members was 1.85 and 1.06 times the rate of urbanized-but-not-metro and metro, respectively.

**Figure 3:**
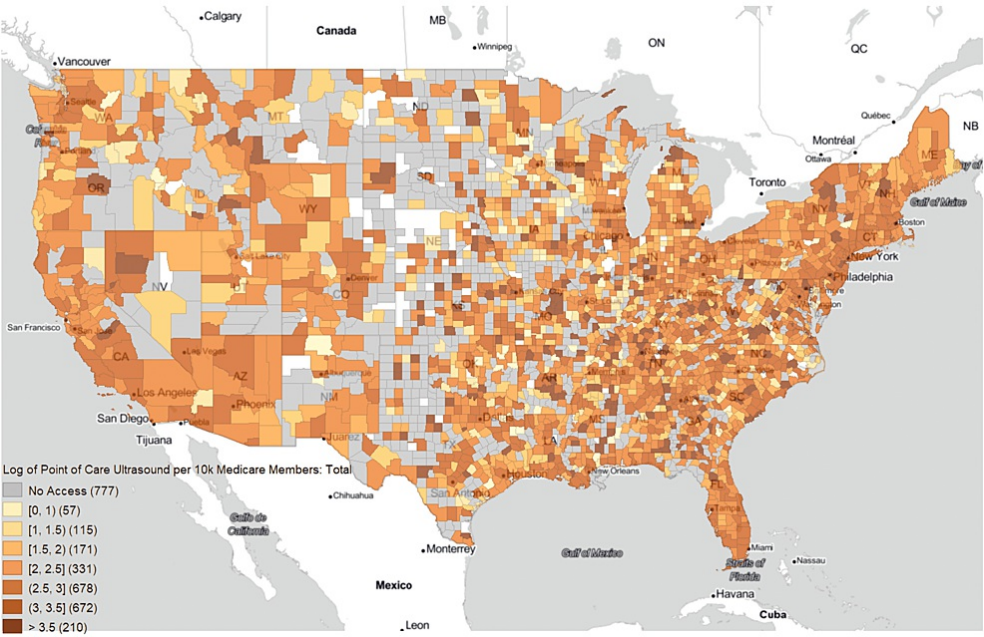
Choropleth map of log-scaled average yearly point-of-care ultrasound uses per 10k Medicare members Counties without access are in gray. Counties excluded from analysis are in white.

The log-scaled yearly average POCUS use per 10,000 Medicare members is shown in Figure 3. Counties without access and counties excluded from analysis are in gray and white, respectively. The highest rates of POCUS were consistently contiguous with rural counties when scaled to population.

**Figure 4:**
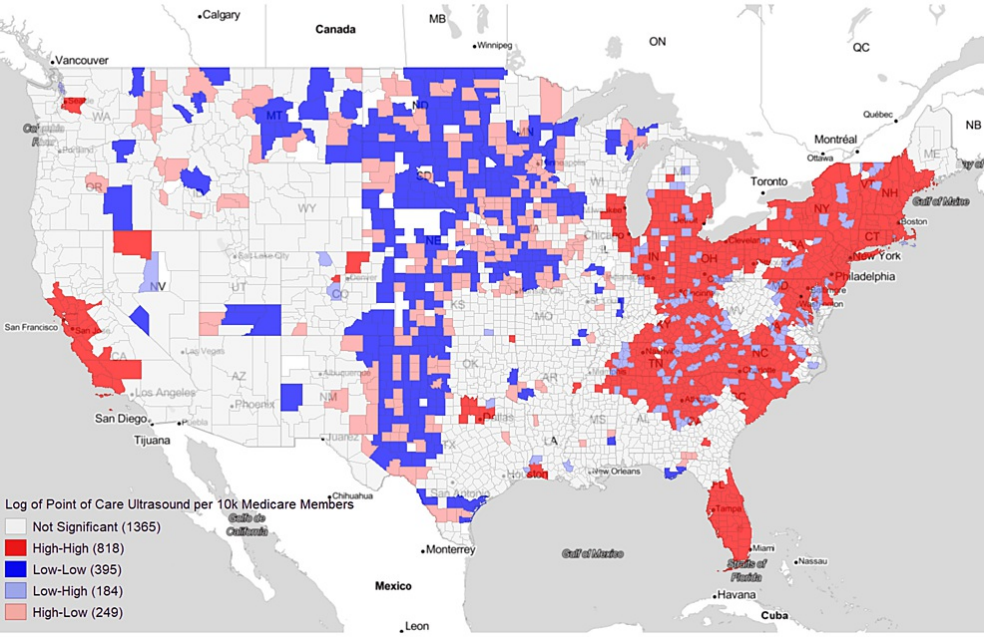
Moran’s I plot of log-transformed POC ultrasounds per 10k Medicare members per year Counties excluded from analysis are in white. POC: point of care

Moran’s I plot uses the log-scaled yearly average POCUS use per 10k Medicare members as shown in Figure 4. Hotspots of POCUS were in South, the Bay Area of California, and the Northeast, with a median of 953.04 POCUS per 10k Medicare members. Coldspot areas were seen mostly in the Great Plains and Midwest regions (median of 0 POCUS per 10k Medicare members per year). Spatial outliers were scattered within hotspot and coldspot clusters. Low-High outliers mostly correspond with the rural and urbanized areas surrounded by metro counties (median of 4.07 POCUS per 10k Medicare members per year) (Figure 1). High-Low outliers mostly corresponded to the metro and urbanized areas surrounded by rural counties (median of 1029.7 POCUS per 10k Medicare members per year) (Figure 1).

Table 1 consists of a 39-variable ANOVA of the High-High, Low-Low, Low-High, and High-Low Moran’s I clusters visualized in Figure 4 of log-scaled average yearly POCUS per 10k Medicare members. Access in hotspot (High-High) clusters, when compared to coldspot (Low-Low) clusters was statistically significant (p < 0.05) for greater percentages of Black populations, (5.18% vs. 2.38% of Medicare members), less Hispanic populations (1.12% vs. 1.49% of Medicare members), denser populations (143.44 vs. 8.35 people per square mile), more urbanization (3.50 vs. 7.12 RUC), higher likelihood of having a college education (22.55% vs. 19.3%), higher likelihood of having an emergency department (ED) visit (716.8 to 611.6 ED visits per 1k Medicare members), greater proportions of obese populations (18.4% vs. 12.6%), and a lower likelihood of being uninsured (8.97% vs. 11.8%).

**Table 1:** ANOVA analysis of Moran’s I POC ultrasound classifications from Figure 3 GED: General Educational Development; POC: point of care; COPD: chronic obstructive pulmonary disease

ANOVA Analysis of POC Ultrasound Clusters									
Cluster	High-High		Low-Low		Low-High		High-Low		P-value, p < 0.05
Counties per Cluster	818		395		184		249		
Demographic Variable	Median	IQR	Median	IQR	Median	IQR	Median	IQR	
Average Medicare Age	71	2	73.2	2	70.6	2	72.2	1.8	6.34 x 10^-104^
% Male	45.49	2.14	46.48	2.65	46.75	2.69	45.7	2.5	2.63 x 10^-23^
% White	89.8	14.04	90.7	9.34	90.88	12.27	92.14	7.61	7.96 x 10^-2^
% Black	5.18	10.04	2.38	3.66	5.18	11.08	1.53	3.21	1.18 x 10^-33^
% Hispanic	1.12	1.68	1.49	3.17	0.86	1.01	1.69	2.78	6.11 x 10^-19^
% Other Race	2.64	1.95	3.85	2.7	1.99	1.49	3.14	1.67	3.05 x 10^-14^
Population Density: Only Medicare Members	30.29	58.8	1.64	3.35	12.44	10.17	6.29	10.32	2.06 x 10^-7^
Population Density: All People	143.44	324.16	8.35	14.69	54.71	48.71	31.62	52.85	1.59 x 10^-6^
% Poverty	14.07	7.07	12.58	5.18	16.11	7.24	12.36	4.66	1.12 x 10^-11^
Median Household Income	51589.7	16523.1	50101.6	11637.7	46322.6	13569.55	53128	10242.4	1.25 x 10^-15^
Unemployment	4.48	1.32	3.44	1.54	4.89	1.69	3.36	1.26	4.50 x 10^-67^
Uninsured	8.97	5.99	11.8	9.24	11.02	5.32	9.66	7.02	7.37 x 10^-20^
% Without GED	11.7	6.67	10	7.75	16.2	7.3	8.1	5.6	4.18 x 10^-19^
% With Only GED	33.7	10.78	34.6	5.75	38.7	8.2	31.4	7.4	3.09 x 10^-27^
% Some College	28.8	5.48	33.5	6.8	28.15	5.25	33.9	5.9	3.87 x 10^-93^
% College Degree	22.55	16	19.3	6.2	14.9	7.45	22.4	10.6	1.73 x 10^-33^
Emergency Department Visits per 1000 Beneficiaries	716.8	145.55	611.6	219.9	749.7	172.9	626.4	197.6	2.15 x 10^-51^
% Medicare Alcohol Abuse	2.05	0.7	1.2	1.39	2	0.58	1.7	0.97	7.17 x 10^-76^
% Medicare Drug Abuse	3.21	1.8	1.32	1.35	3.44	2.75	1.97	1.52	5.22 x 10^-108^
% Medicare Tobacco Use	10.4	4.4	7.8	3.2	12.2	4.65	8.4	3.2	1.95 x 10^-75^
% Medicare Arthritis	33.45	4.84	30.12	7.54	33.63	5.51	30.58	6.82	8.49 x 10^-27^
% Medicare Diabetes	28.19	4.64	23.76	5.13	29.34	4.38	23.96	4.72	4.29 x 10^-61^
% Medicare Depression	18.82	3.98	15.52	4.08	19.27	4.27	17.46	3.86	2.42 x 10^-67^
% Medicare Ischemic Heart Disease	27.32	5.67	25.9	8.17	28.15	6.05	25.3	6.76	6.58 x 10^-13^
% Medicare Hypertension	60.36	6.18	52.2	11.53	61.22	7.31	54.22	10.24	7.57 x 10^-103^
% Medicare Heart Failure	14.11	3.36	14.56	4.85	15.01	3.21	13.68	4.18	2.66 x 10^-8^
% Medicare Chronic Kidney Disease	23.3	4.25	19.46	4.45	23.69	3.87	20.2	4.2	2.34 x 10^-80^
% Medicare Obesity	18.4	6.2	12.6	4.8	18.8	6.6	14.4	4.8	1.26 x 10^-106^
% Medicare Osteoporosis	5.78	1.67	5.14	2.14	4.82	1.64	5.74	2.18	2.93 x 10^-17^
% Medicare Stroke	3.74	0.82	2.62	0.95	3.5	0.88	2.82	0.86	5.89 x 10^-124^
% Medicare COPD	13.03	4.46	10.64	3.31	14.72	4.38	11.26	3.42	1.78 x 10^-46^
Log of Point-of-Care Ultrasound per 10k Medicare Members: Total	2.98	0.55	-0.04	0	0.7	1.65	3.01	0.74	0
Point-of-Care Ultrasound per 10k Medicare Members: Total	953.04	1323.7	0	0	4.07	39.24	1029.7	1683.17	1.30 x 10^-93^
Point-of-Care Ultrasound per 10k Medicare Members: Diagnostic Exam	737.94	980.37	0	0	0	26.77	808.47	1344.4	7.14 x 10^-89^
Point-of-Care Ultrasound per 10k Medicare Members: Guided Procedure	200.93	350.18	0	0	0	4.94	196.9	484.63	5.72 x 10^-58^
Demographic Variable	Mean	SD	Mean	SD	Mean	SD	Mean	SD	
Rural-Urban Continuum Code (integer 1-9, 1=most urban, metro<=3)	3.5	2.39	7.12	2.11	5.02	2.81	5.32	2.44	1.13 x 10^-115^
Metro (binary value 0,1)	0.58	0.49	0.1	0.31	0.38	0.49	0.32	0.47	1.47 x 10^-60^
Urbanized, Not Metro (binary value 0,1)	0.35	0.48	0.42	0.49	0.38	0.49	0.49	0.5	1.75 x 10^-3^
Rural (binary value 0,1)	0.07	0.25	0.48	0.5	0.24	0.43	0.19	0.4	1.58 x 10^-66^

## Discussion

The disparity in healthcare outcomes between rural and urban populations has been well documented. Rural populations tend to have higher rates of chronic disease and premature deaths while also having diminished access to healthcare, especially to specialty healthcare services [20-23]. Our analysis shows a clear disparity between absolute access to ultrasound between rural, urbanized, and metro areas but also in the relative use rates of POCUS, which is most clearly visualized in Figure 4. These two measures likely influence each other, as the lack of access to ultrasound will impact the rates of its use within a geographic region. Coldspot, low access areas, were significantly more rural, poor, and uninsured but with less chronic comorbidities than the wealthier, urban populations (Table 1). Most studies have found an increase in chronic conditions among rural populations, which conflicts with our findings. Both can be true, however, as a limitation of our study’s Medicare-only population is that it may not be fully reflective of the communities at large. But, as Medicare communities are exceedingly large and uniquely vulnerable populations, the analysis is still valuable. Interestingly, our analysis shows higher relative rates of POCUS in High-Low counties than in High-High hotspots or clusters. This is further supported after removing all counties with no access - of the remaining counties, 94% of urbanized-but-not-metro counties had POCUS access. This likely indicates that these High-Low regions are urbanized-but-not-metro counties that are servicing surrounding counties that have lower access to POCUS.

Since 2010, over 100 rural hospitals have closed, exacerbating the limited access to care for rural populations [20]. Reasons for closure have included cutbacks in Medicare reimbursement, reduced funding, and imminent deadlines for instituting costly electronic medical records [13]. While operating margins of metro hospitals have increased in recent years, operating margins of rural hospitals have steadily decreased [24]. Though ultrasound is considered an inexpensive tool, the initial cost of purchase for an ultrasound system is high. The average price of ultrasound systems is about $115,000, with low-end systems costing $25,000 and high-end systems being upwards of $250,000 [25]. This represents a major purchase for small rural hospitals that already have decreased financial performance. While ultrasound systems may be costly to acquire, they ultimately save money in the long run by eliminating additional diagnostic testing and costly workup. On average, a single use of POCUS that changes a patient’s management will save $181.63 of potentially negative-margin additional CMS-reimbursable procedures or tests for the hospital [26]. This translates to $1134.31 of additional testing saved for privately insured patients and $2826.31 saved for out-of-network or uninsured patients [26].

Affiliation of rural hospitals with large healthcare systems may also be contributing to reduced ultrasound access in rural populations. Many small rural hospitals have affiliated themselves with large healthcare systems to improve financial performance and avoid closure. However, there is a decrease in on-site imaging studies performed once a hospital affiliates [24]. These changes have increased disparities in obstetrical care with the loss of hospital-based obstetric services [27]. Limited ultrasound access affects maternal and neonatal outcomes in rural communities due to increased distances required to travel, which creates financial and logistical barriers [28]. This may be due to health systems dropping duplicative services or equipment that was costly to maintain. Imaging studies may additionally be dropped since they could be accessed at tertiary facilities within the healthcare system, where specialists could provide both imaging and other services as needed [24]. Interestingly, rates of visits to rural EDs have significantly increased over time, which may indicate poor accessibility to primary care as well as the increased burden of illness that rural populations experience [29]. A useful application of POCUS is in the ED, and this further emphasizes the importance of access to ultrasound in rural EDs. Another barrier is the lack of supporting services for ultrasound use, including radiology. The lack of radiology services is a large driver in transfers from rural EDs to urban EDs [30].

Lack of training in use of ultrasound may furthermore provide an obstacle for access to ultrasound in rural areas. A study in Missouri found that many of the surveyed hospitals indicated a lack of training as an impediment to greater use of POC ultrasonography in their EDs [9]. Another study found that the primary reason cited for not implementing emergency physician-performed ultrasonography was the lack of emergency physician training [31]. This lack of training is widely known, and, as a result, many medical schools have implemented ultrasound into their curriculum [32]. Ninety core clinical milestones in ultrasound use have even been established for all graduating medical students to serve as a curriculum guideline [32]. Remote education is another option for training, and a systematic review from 2019 on global ultrasound access showed that ultrasound techniques can be adequately taught and monitored remotely via telemedicine [33].

To increase access to ultrasound in rural communities, telemedicine options have expanded as an alternative. A group in Canada recently published results from the implementation of a “telerobotic ultrasound clinic.” Sonographers remotely operated the robotic ultrasound machine, and radiologists at academic centers remotely interpreted the images. They found that 70% of examinations were sufficient for diagnosis, and 95% of patients would undergo another robotic ultrasound examination [34]. New companies, such as Butterfly Network (Guilford, CT, USA), aim to expand ultrasound access with their handheld device that is accompanied by integrated storage and workflow processes for a much more affordable price. Another option for expanding access is a donation of refurbished ultrasound machines to rural hospitals and clinics.

There have been many previous reports of limited access to ultrasound in rural settings as well as explorations of the barriers to access. Our analysis is the first to examine the geospatial disparities in POCUS access at the county level across the United States, further confirming the disparity between rural and urban POCUS access. We found that populations that are most affected by this lack of access are those that are less educated, have lower income, or are uninsured. This multifactorial problem of limited POCUS access has no easy solution with many financial, training, and resource barriers, though there are increasing numbers of telemedicine initiatives. With the diagnostic importance and long-term monetary benefits of POCUS, increasing access to ultrasound in rural areas has the potential to significantly benefit these populations.

## Conclusions

While ultrasound is an inexpensive, noninvasive, and multifunctional tool, there exists a large disparity in use between rural and urban areas in the United States. Our analysis shows that only 38.84% of rural counties (compared to 88.56% of metro counties) had any access to POCUS in 2015-2019. This difference in ultrasound access has a multitude of contributing factors; however, we speculate financial difficulties and lack of training in ultrasound to be key factors. Future research should be conducted into the reasons for differing access to ultrasound between rural and metro areas. Ultrasound has wide-reaching utility in healthcare, can enhance clinical outcomes, and save money for hospitals. With these factors in mind, it is invaluable that increased emphasis and initiatives be placed on access to ultrasound in rural areas.
